# Study on the Modification and Minding Mechanism of Bongkrekic Acid Aptamers for Food Safety

**DOI:** 10.3390/foods15101663

**Published:** 2026-05-10

**Authors:** Xufei Sun, Haoyu Yang, Yunzhe Zhang, Xin Lu, Hui Xu, Qinghai Sheng, Congyan Qi, Wei Zhang

**Affiliations:** 1 College of Food Science and Technology, Hebei Agricultural University, Baoding 071001, China; sxf15031187527@163.com (X.S.); 13290522600@163.com (Q.S.); cyqi@hebau.edu.cn (C.Q.); 2 Department of Physical Education and Sport, Hebei Agricultural University, Baoding 071001, China; y202604@126.com; 3 Experiment and Practice Training Center, Hebei Agricultural University, Baoding 071001, China; mickey2170031@126.com; 4 College of Science and Technology, Hebei Agricultural University, Cangzhou 061100, China; lu201709@126.com (X.L.); yukyhaoyidian@126.com (H.X.); 5 Hebei Provincial Key Laboratory of Analysis and Control for Zoonoses Microbial, College of Life Sciences, Hebei Agricultural University, Baoding 071001, China

**Keywords:** bongkrekic acid, SELEX, aptamer, food safety

## Abstract

Bongkrekic acid is a lethal mitochondrial toxin produced by *Burkholderia gladioli pathovar cocovenenans*, posing severe threats to food safety due to their high stability and the lack of effective antidotes. Developing specific, high-affinity recognition elements is crucial to overcoming the limitations of current BA detection methods in food matrices, and thereby safeguarding food safety and public health. In this study, we report for the first time the selection and remodelling of a DNA aptamer with high affinity for BA, which could be used as a promising recognition tool for sensitive BA detection in food. Integrating isothermal titration calorimetry, molecular docking, and molecular dynamics simulations revealed that the binding of BA to F3-1 follows an induced-fit mechanism. This study is the first to report a DNA aptamer with nanomolar affinity for BA, clarify its underlying binding mechanism, and provide a reliable recognition element for sensitive and specific BA detection in food samples.

## 1. Introduction

Food safety remains a critical global challenge, with microbial toxins representing a persistent threat to public health. Among these, bongkrekic acid (BA), a lethal mitochondrial toxin produced by *Burkholderia gladioli pathovar cocovenenans*, stands out for its extreme toxicity and chemical stability [1,2].

BA is mainly found in cereal products (fermented corn flour, glutinous rice ball with corn, corn starch, etc.), tremella, black fungus, coconut and its products, as well as potato products (potato vermicelli, sweet potato starch, mountain yam starch, etc.) [3], leading to an extremely high mortality rate of 40% to 100% due to the lack of specific antidotes [4]. As a mitochondrial toxin, bongkrekic acid is characterized by high toxicity and high mortality rate, causing severe damage to vital human organs, including the liver, kidneys, heart, and brain. Being odorless, tasteless, and highly thermostable, it cannot be eliminated by ordinary cooking methods. Therefore, its presence in contaminated food is difficult to detect [5]. The urgency for effective monitoring is underscored by recurring outbreaks, which call for rapid, on-site detection methods to prevent contaminated products from reaching consumers [2,6,7,8,9].

Conventional techniques for BA detection, such as liquid chromatography-tandem mass spectrometry (LC-MS/MS), offer high accuracy but are hampered by their reliance on sophisticated instrumentation, skilled personnel, and lengthy procedures, making them unsuitable for rapid screening [1,10]. Immunoassays provide a portable alternative, yet the high toxicity of BA poses significant challenges in generating high-quality antibodies [11,12]. Among the common immunoassays for rapid detection of BA, GICA features high sensitivity, low cost, simple operation and good specificity, making it suitable for large-scale food safety screening. However, it suffers from poor particle size uniformity, unstable immune labeling and only allows qualitative analysis. ELISA exhibits satisfactory sensitivity and repeatability, but involves cumbersome procedures and relatively low detection speed. FICA enables rapid detection, simple operation and on-site testing, yet is susceptible to fluorescence interference, accompanied by high false-positive rates and limited quantitative range. Thus, there is a pressing need for novel recognition probes that combine high affinity, stability, and ease of production [13].

DNA aptamers, single-stranded oligonucleotides selected through the Systematic Evolution of Ligands by Exponential Enrichment (SELEX), have emerged as promising molecular recognition elements [14]. Their advantages include synthetic accessibility, excellent stability, and the capacity to undergo reversible conformational changes upon target binding [15,16]. Although aptamers have been successfully generated targeting various mycotoxins, including ochratoxin A and aflatoxin B1, no BA-specific aptamer has been reported to date, creating a critical gap in the development of aptamer-based biosensors for this hazardous mycotoxin [17,18,19,20]. A profound understanding of the aptamer-ligand interaction mechanism is crucial for guiding post-SELEX remodeling and biosensor design. However, the structural basis and binding dynamics for most small-molecule-binding DNA aptamers remain poorly characterized, often due to the scarcity of high-resolution structural data. This lack of mechanistic insight represents a major bottleneck in the rational engineering of aptamers with enhanced performance.

Therefore, this study aims to screen specific DNA aptamers against BA via the SELEX technique and perform remodelling to further improve their affinity. On this basis, the interaction mechanism and molecular recognition pattern between aptamers and BA are systematically analyzed using isothermal titration calorimetry, molecular docking, and molecular dynamics simulation. Ultimately, highly specific recognition elements applicable to food safety will be obtained, providing reliable molecular tools and theoretical support for the subsequent development of rapid detection techniques for bongkrekic acid.

## 2. Materials and Methods

### 2.1. Reagents and Apparatus

All toxins were purchased from Pribolab Co., Ltd. (Qingdao, China). All the oligonucleotide sequences were purified by HPLC and supplied by Sangon Biotech Co., Ltd. (Shanghai, China). The binding buffer (BB, including Tris-HCl, NaCl, KCl, and MgCl_2_) was purchased from Beijing BioDee Biotechnology Co., Ltd. (Beijing, China) The Taq DNA polymerase premixed solution was purchased from ApexBio Technology (Houston, TX, USA). N,N,N’,N’-Tetramethylethylenediamine (TEMED), TBE buffer, and 30% acrylamide solution were purchased from Beijing Solarbio Science & Technology Co., Ltd. (Beijing, China). Ammonium persulphate (AP) was purchased from Beijing Lvyuan Dade Biotechnology Co., Ltd. (Beijing, China). The high-speed Centrifuge (model TG16A) was purchased from Shanghai Lu Xiangyi Centrifuge Instrument Co., Ltd. (Shanghai, China). The thermal cycler (Model TC-5) was purchased from Techne Ltd. A gel imaging system (JY04S-3E) was purchased from Beijing Junyi Dongfang Electrophoresis Equipment Co., Ltd. (Beijing, China) Nano ITC was obtained from TA Instruments Ltd. (New Castle, DE, USA). The F-320 spectrofluorometer was purchased from Qingdao Lubo Weiye Environmental Technology Co., Ltd. (Qingdao, China).

The DNA sequence required for this experiment is as follows:

DNA library: ATAGGAGTCACGACGACCAGAAN_40_TATGTGCGTCTACCTCTTGACTAAT
Primer I: ATAGGAGTCACGACGACCAGAA
Primer II: ATTAGTCAAGAGGTAGACGCACATA.

### 2.2. Aptamer Selection

#### 2.2.1. Aptamer Screening Process

Dilute BA with methanol to a final concentration of 10 μg/mL as the working solution. The initial ssDNA library (20 μL, 100 μM) was dissolved in 400 μL of binding buffer and subjected to the following heat treatments in sequence: incubation in a metal bath at 95 °C for 10 min, and incubation at 25 °C for 15 min. Subsequently, the diluted BA working solution was mixed with the treated initial library and incubated at 37 °C for 45 min (binding). After incubation, centrifuge the mixed solution at 4 °C and 15,656 g for 15 min. Discard the supernatant, then wash the sediment twice with binding buffer. Finally, dissolve the precipitate in deionized water (ddH_2_O) and use this solution as a template for asymmetric PCR amplification. The resulting amplification products will serve as a secondary library for the next round of screening. The above screening steps are repeated. At the 10th round of selection, counterselection was performed using interfering toxins to improve the specificity of the obtained aptamers. The amplification program and system of asymmetric PCR is detailed in Table 1.

#### 2.2.2. Aptamer Sequence Analysis

A total of 18 rounds of cyclic screening were conducted by the screening steps described in Section 2.2.1. After the screening of the 9th and 18th rounds was completed, the enriched libraries of this round were collected, respectively, for DNA sequencing (BGI Genomics Co., Ltd., Copenhagen, Denmark). Compare and analyze the sequencing results from the two rounds to evaluate the extent of sequence enrichment that occurred during the screening process. We predicted and analyzed the secondary structures of the candidate aptamers using mFold (v3.6). The three-dimensional structure was simulated and predicted using RNAComposer (v1.0) and PyMOL (v3.1).

#### 2.2.3. Aptamer Molecular Docking Simulation

Based on the secondary structure of the selected aptamer, we first used RNAComposer to automatically generate its RNA tertiary structure model [21,22,23]. Subsequently, we performed visualization processing in PyMOL and replaced the uracil (U) in the model with thymine (T) to simulate the state of the DNA aptamer, ultimately generating the tertiary structure of the aptamer. We used AutoDockTools (v4.2.6) for three-dimensional molecular docking simulations to predict the binding mode of the aptamer and BA; we used PyMOL to conduct visual analysis of the docking results and elucidate the interaction mechanism between the two molecules at the molecular level.

#### 2.2.4. Aptamer Affinity Determination

Isothermal titration calorimetry (ITC) is a key technology for studying the thermodynamics of biomolecular interactions and binding affinity. This method uses a highly sensitive microcalorimeter to automatically monitor the thermodynamic curve of the titration process under unlabeled in situ conditions, simultaneously obtaining parameters such as enthalpy change (ΔH), entropy change (ΔS), and affinity constant (Kd). Given that the reliability of ITC in verifying the specific binding activity of small molecules and aptamers has been widely confirmed [24], this study used this technology to perform analysis of the binding affinity of the aptamers screened in Section 2.2.1.

The test concentration of BA was 0.1 mM, and the test concentrations of the aptamers were both 0.01 mM. The initial volume of BA titration was 50 μL (inhaled into the syringe); The initial volume of aptamer titration was 300 μL (added to the sample cell), a total of 20 drops, 2 μL each drop. The titration interval is set to 120 s, and the stirring speed is set to 350 rpm. Control group: The binding buffer was used as the blank control instead of the aptamer solution. All experiments were conducted under a constant temperature of 25 °C.

### 2.3. Aptamer Remodeling

Based on the ITC test results, the aptamers with the best affinity and specificity were selected as the remodeling targets. The binding sites of all the aptamers obtained through screening were respectively replaced with the corresponding bases at the same positions of the remodeling targets, and molecular docking simulations and ITC affinity determinations were conducted to select the optimal aptamer. Molecular dynamics simulations were then performed on this aptamer to elucidate its binding mechanism with BA further.

#### 2.3.1. Remoded Aptamer Molecular Docking Simulation

Refer to Section 2.2.3.

#### 2.3.2. Remoded Aptamer Affinity Determination

Refer to Section 2.2.4.

#### 2.3.3. Molecular Dynamics Simulation Is Used to Analyze the Dynamic Process of Aptamer Binding to BA

MD simulations can precisely evaluate the binding affinity and mode of aptamers with ligands, dynamically visualizing the entire induction-binding kinetic process. This technique facilitates a deeper understanding of the conformational changes, atomic relative displacements, and evolution of interaction forces during aptamer binding, thereby predicting the most stable spatial conformation of the complex [25,26]. Therefore, applying MD simulations to investigate the interaction between the aptamer and BA can clearly reveal the dynamic details and molecular mechanisms of their binding.

##### Calculation of Root Mean Square Deviation During the Aptamer Binding Process

Root-mean-square deviation (RMSD) is a crucial parameter for describing the difference between simulated conformations and crystal structures. By calculating the RMSD value, one can determine the similarity between a simulated conformation at a given moment and the target conformation, representing the sum of deviations for all atoms. Each frame has an RMSD value, making it a crucial basis for assessing system stability. The RMSD value of a nucleic acid aptamer at a specific time can be obtained using the following formula:

$$RMSD\alpha (tj)=\sqrt{\frac{{\sum_{\alpha =1}^{N_{\alpha }}\left(r_{\alpha }\left(tj\right)-(r_{\alpha })\right)}^{2}}{N_{\alpha }}}$$

By calculating the RMSD values of the aptamer–BA complex system over time, one can estimate the conformational changes in the aptamer before and after binding to BA.

##### Distance Changes Between Bases G18, A20, G36, and G37 on the Aptamer

Through molecular docking, we identified the bases G18, A20, G36, and G37 as the binding sites for BA. Therefore, we focused on investigating how the distances between these bases change over time, using this as a reference standard for structural stability and conformational changes.

##### Determination of Hydrogen Bond Counts Formed Between Bases G18, A20, G36, and G37 on the Aptamer and BA

Hydrogen bonds, as one of the primary contributing forces in the binding between BA and aptamers, play a crucial role in the structural stability of the aptamer–BA complex. Therefore, the number of hydrogen bonds formed between G18, A20, G36, and G37 with BA was investigated over time.

### 2.4. Aptamer Application Performance Evaluation

6-FAM-labeled F3-1 aptamer and BHQ1-labeled complementary strand (cDNA) were synthesized and purified by HPLC. Standard stock solutions of BA (1 mg/mL) were prepared in methanol and serially diluted with binding buffer (BB) to final concentrations ranging from 0 pg/mL to 100 ng/mL. Interfering toxins, including aflatoxin B1 (AFB1), tetrodotoxin (TTX), and Toxoflavin (TF), were prepared as 1 mg/mL stock solutions in appropriate solvents.

For LOD determination: 6-FAM-labeled F3-1 aptamer (10 μM, 10 μL), BHQ1-labeled complementary strand (10 μM, 10 μL), and MgCl_2_ (100 mM, 2 μL) were added to a centrifuge tube, followed by BB to adjust the initial volume to 100 μL. The mixture was incubated at 37 °C for 45 min to allow full hybridization. Subsequently, 100 μL of BA standard solutions with different concentrations (0 pg/mL, 700 pg/mL, 800 pg/mL, 900 pg/mL, 1 ng/mL, 2 ng/mL, 10 ng/mL, 50 ng/mL, 100 ng/mL) were added to the reaction system, mixed thoroughly, and incubated at 37 °C for 2 h. Finally, Tris-HCl buffer (10 mM, pH 7.4) was added to adjust the total volume to 200 μL. Fluorescence intensity was measured using an F-320 spectrofluorometer with excitation at 488 nm and emission at 520 nm. The relative fluorescence intensity ratio ((F − F_0_)/F_0_) was calculated, where F is the fluorescence intensity of the sample containing BA and F0 is the fluorescence intensity of the blank control (without BA). A standard curve was plotted with LgC_BA_ as the abscissa, and (F − F_0_)/F_0_ as the ordinate, and the LOD was calculated based on the 3σ rule (σ is the standard deviation of blank measurements).

For specificity analysis: Five experimental groups were designed: blank group (no toxin), BA group (BA concentration = 1 ng/mL), interfering toxin groups (AFB1, TTX, TF; each concentration = 10 ng/mL), and mixed toxin group (BA, AFB1, TTX, TF; each concentration = 1 ng/mL). The reaction system and incubation conditions were consistent with those for LOD determination. Fluorescence intensity at 520 nm was measured for each group, and the relative fluorescence intensity was calculated to evaluate the specificity of the aptamer.

## 3. Results and Discussion

### 3.1. Analysis of Aptamer Screening Results

#### 3.1.1. Aptamer Sequence Analysis

**Figure 1:**
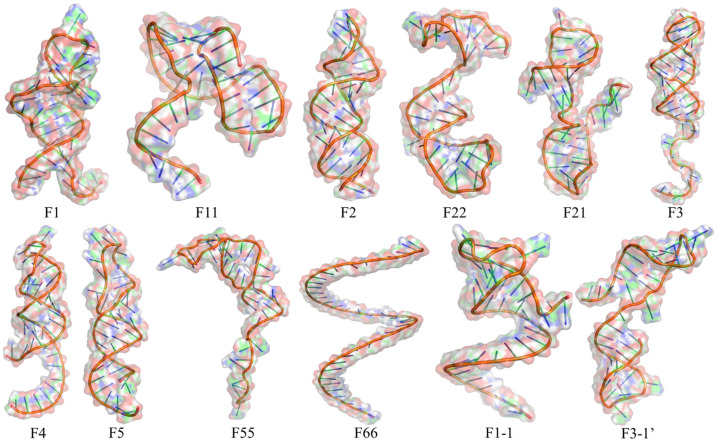
Tertiary structure diagram of aptamers.

A total of 12 DNA aptamers were screened (F1, F11, F2, F22, F21, F3, F4, F5, F55, F66, F1-1, F3-1′), with detailed information presented in Table 2. Their secondary structures are shown in Supplementary Figure S1. All 12 BA-binding aptamers featured a canonical stem-loop structural motif, which is composed of double-stranded stem regions formed by complementary base pairing and single-stranded loop/bulge domains. The double-stranded stems serve as the structural skeleton to maintain the conformational stability of aptamers, while the single-stranded loops/bulges act as the potential functional domains for molecular recognition and binding, which are the typical structural characteristics of DNA aptamers targeting small-molecule ligands. The tertiary structures are shown in Figure 1.

As can be seen from Figure 1, only F66 exhibits an unfolded linear conformation (single helix state), while the remaining aptamers fold and form stem-loop structures. It is worth noting that the positions of the stem-loop structures formed by some aptamers in three-dimensional structures differ from the positions predicted in their secondary structures. The main reason for this discrepancy lies in the fact that secondary structure prediction algorithms (such as the method used in this study) primarily rely on double-stranded stem regions and unpaired loop regions formed by base pairing (particularly Watson–Crick pairing) to find the structural conformation with the lowest global free energy. However, the actual three-dimensional structural folding process is influenced by many more factors, including steric hindrance, long-range interactions, precise geometric constraints, and environmental effects such as solvation. Therefore, inconsistencies between secondary structure predictions and three-dimensional structure models at stem-ring positions are common, and sometimes even quite significant.

**Figure 2:**
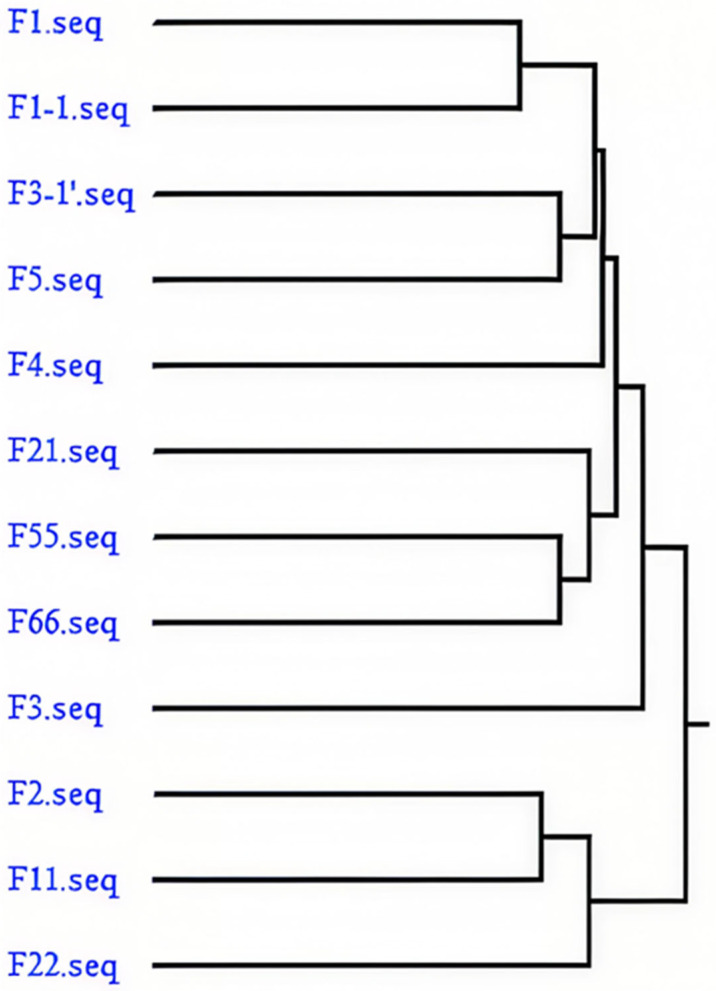
Analysis of family affinity.

DNAMAN homology analysis clearly revealed that the 12 candidate aptamer sequences could be classified into three homologous families and one independent sequence (Figure 2): Group 1 consisted of F1 and F1-1, which exhibited the shortest branch and the highest homology, indicating enrichment and variation from the same original sequence. Group 2 represented the core enriched family, including F3-1′, F5, F4, F21, F55, F66, and F3, with close phylogenetic relationships among members, especially high homology between F3-1′ and F5 as well as between F66 and F55, accounting for the largest proportion and representing preferential enrichment during SELEX. Group 3 comprised F2 and F11 with high homology, forming another independent sequence family. In contrast, F22 formed an individual branch with the most distant phylogenetic relationship to all other sequences, representing a unique sequence type with potentially distinct binding patterns or structural characteristics. Based on this phylogenetic analysis, representative sequences from the core family (such as F3 and F66) and the independent sequence F22 were selected for subsequent affinity determination to cover different homologous clusters and avoid redundant testing of highly similar sequences. Overall, such clustering results reflected the sequence enrichment preference during SELEX screening and provided a solid basis for the selection of representative aptamers and further functional characterization.

#### 3.1.2. Analysis of Simulation Results of Aptamer Molecular Docking

**Figure 3:**
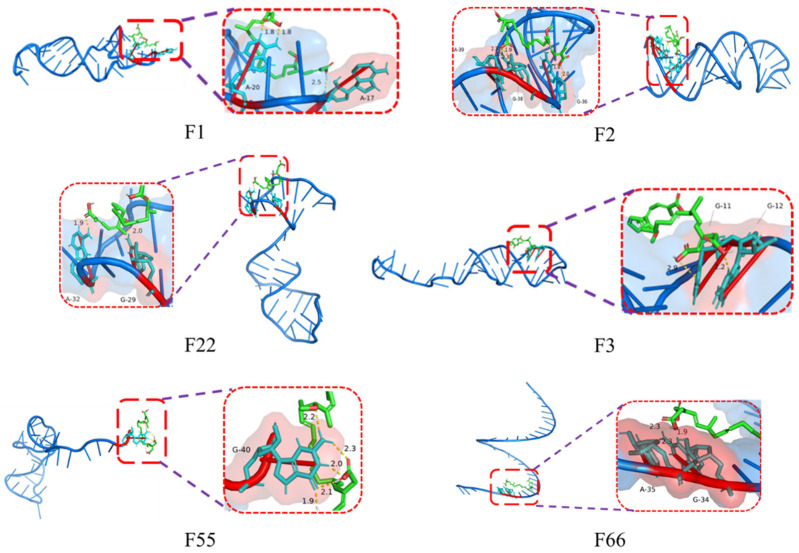
Molecular docking simulation results.

Molecular docking simulations revealed that only aptamers F1, F2, F22, F3, F55, and F66 exhibit spontaneous binding interactions with BA, while binding for all other aptamers is non-spontaneous. As shown in Figure 3, the base-pairing sites between DNA aptamers and BA are clearly visible: F1 binds at A17 and A20; F2 binds at G36 and G38, A39; F22 binds at G29 and A32; F3 binds at G11 and G12; F55 binds at G40; F66 binds at G34 and A35. This indicates that BA primarily forms specific bonds with the adenine (A) and guanine (G) bases in the aptamer. The reason is likely due to the molecular structure of BA and its interaction characteristics with the bases. This also provides a theoretical foundation for future remodeling of BA aptamers.

#### 3.1.3. Analysis of Aptamer Affinity Determination

Affinity analysis was performed on the six adapters obtained in Section 3.1.2 using ITC. Test results were visualized using Origin software. Table 3 presents the test data results fitted based on the Independent model, yielding corresponding parameters including dissociation constant Kd, stoichiometric ratio n, enthalpy change ΔH, and entropy change ΔS. The Gibbs free energy change ΔG was calculated according to the following formula.

ΔG = ΔH − T·ΔS

**Figure 4:**
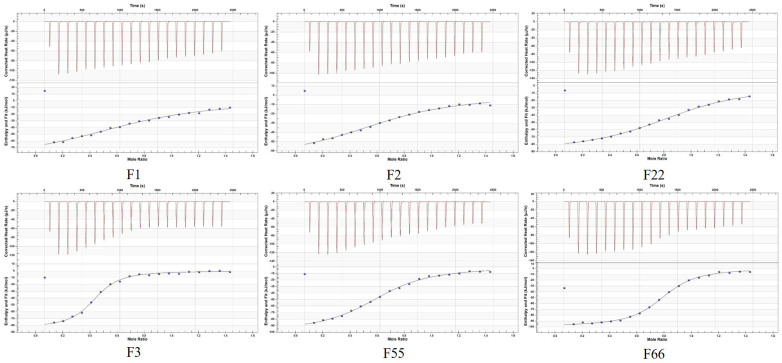
The ITC titration curve of the interaction between BA and aptamers.

The upper half of Figure 4 shows the heat power (μJ/s) generated during each titration over time. A distinct exothermic peak (negative value) is observed at the onset of titration, indicating binding between the aptamer and BA. As titration progresses, the peak height gradually decreases. The relatively smooth curve suggests no significant non-specific aggregation during binding. The lower panel of Figure 4 displays the molar binding enthalpy (kJ/mol) fitted to the molar ratio using the Independent model. Only the curves for F3, F55, and F66 exhibit an “S”-shaped profile, indicating specific binding. Among these, the curves for F3 and F66 are steeper than those for other aptamers, demonstrating stronger binding affinity.

As shown in Table 3, the BA titration process of aptamers F1, F2, F3, F22, F55, and F66 exhibits a noticeable heat change, indicating an exothermic reaction. This observation reveals the binding effect between BA and aptamers, which is consistent with the results presented in Figure 4. The Kd value (dissociation constant) reflects the stability of the aptamer–BA complex, with smaller values indicating stronger affinity. Therefore, in terms of Kd values, F66 and F3 are the most efficient aptamers, F55 and F22 have moderate affinity, and F1 and F2 have the weakest affinity. The *n* value of F22 was the highest, reaching 0.915, which was most consistent with the 1:1 binding model, proving that it had the best binding specificity.

### 3.2. Analysis of Aptamer Remodeling Results

Based on the above findings, aptamers F66 and F3 exhibit high affinity, while aptamer F22 demonstrates high specificity. Therefore, aptamer remodeling primarily involved base substitutions based on these three aptamers. The binding sites of all screened aptamers were replaced with the corresponding bases at the same positions in F22, F3, and F66, ultimately yielding 24 optimized aptamers. Refer to Table 2 for details F3-1 to F22-8.

#### 3.2.1. Analysis of Aptamer Molecular Docking Simulation Results

**Figure 5:**
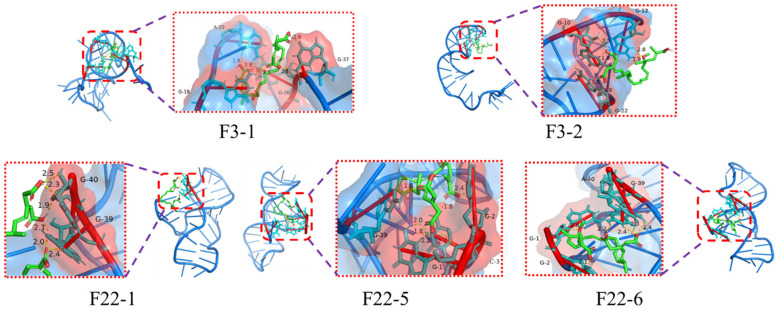
Molecular docking simulation diagram of the remodeled aptamers with BA.

**Figure 6:**
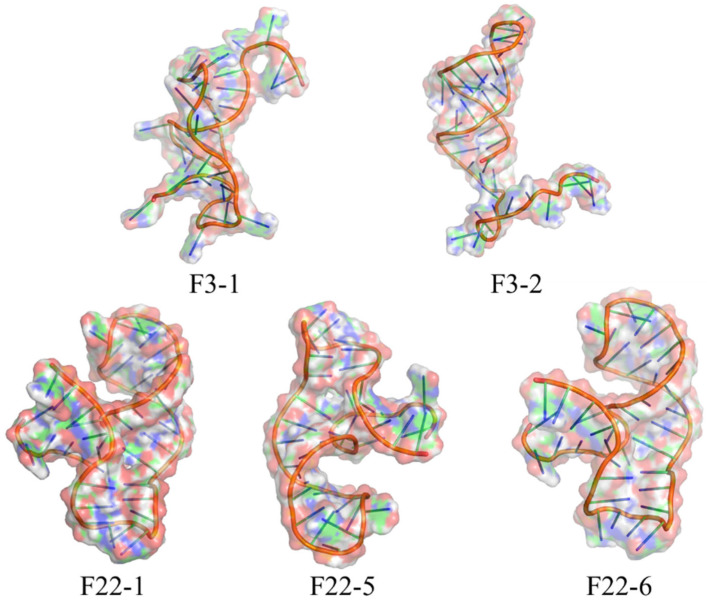
Tertiary structure of the five remodeled aptamers.

Molecular docking simulations were performed between the remodeled aptamers from Section 3.2 and BA to identify their binding sites. The results revealed that only aptamers F3-1, F3-2, F22-1, F22-5, and F22-6 exhibited spontaneous binding interactions with BA, whereas the binding of all other aptamers to BA was non-spontaneous (Figure 5). Analysis of the binding site bases and whether the binding reactions proceeded spontaneously revealed that only F3-1, F3-2, F22-1, F22-5, and F22-6 exhibited spontaneous binding reactions. The binding sites for F3-1 are G18, A20, G36, and G37; for F3-2, G10, G12, and G22; for F22-1, G39 and G40; F22-5 binds at G1, G2, C3, and G39; F22-6 binds at G1, G2, G39, and A40. Notably, F3-1 and F3-2 differ only at the 32nd base yet exhibit entirely distinct binding sites. In contrast, F22-5 and F22-6, though differing only at the 32nd base, exhibit highly similar binding sites. Its 3D structure is shown in Figure 6. From the figure, it can be seen that the structures of F22-5 and F22-6 are very similar. We hypothesize this discrepancy arises because the tertiary structures of F3-1 and F3-2 diverge significantly, whereas those of F22-5 and F22-6 are extremely similar.

#### 3.2.2. Aptamer Affinity Analysis

Based on the results of the molecular docking simulation in Section 3.2.1, the affinities of the five obtained aptamers were determined, and the results are as follows.

**Figure 7:**
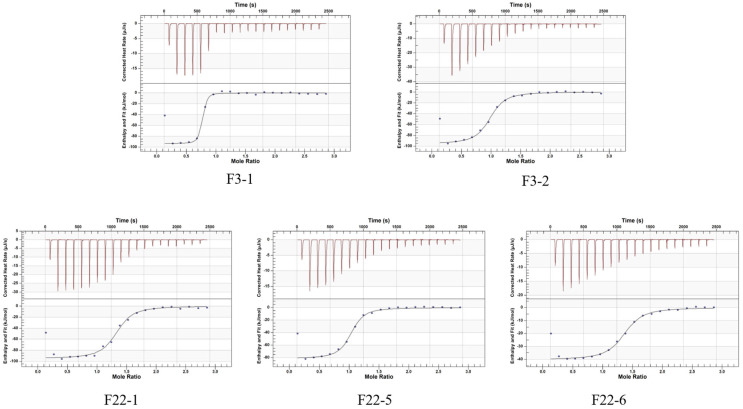
The ITC titration curve of the interaction between BA and the remodeled aptamers.

The upper panel of Figure 7 shows a distinct exothermic peak during the initial titration, indicating binding between the aptamer and BA. As titration progresses, the peak height gradually decreases and approaches zero, indicating that the binding sites are gradually reaching saturation. The titration curves for all five aptamers are relatively smooth with no significant noise, indicating a stable binding process without noticeable non-specific binding or aggregation. The lower panel shows that the fitting curves for all five aptamers exhibit a typical S-shaped profile, confirming specific binding. The curves subsequently flattened near a molar ratio of “1”, verifying that binding reached saturation. Notably, the curves for F3-1 and F22-5 were steeper compared to the other aptamers, demonstrating stronger binding affinity.

According to Table 4, it can be seen that the Kd values from small to large are: F3-1 (61.9 nM) < F22-5 (598.8 nM) < F22-1 (839 nM) < F3-2 (891.8 nM) < F22-6 (932.2 nM). The affinity of F3-1 reached 61.9 nM, which was 144 times higher than that of F3. A very strong, specific interaction between BA and F3-1 was demonstrated.

#### 3.2.3. Analysis of Molecular Dynamics Simulation Results

##### Analysis of the RMSD Value of the Aptamer–BA Complex over Time

**Figure 8:**
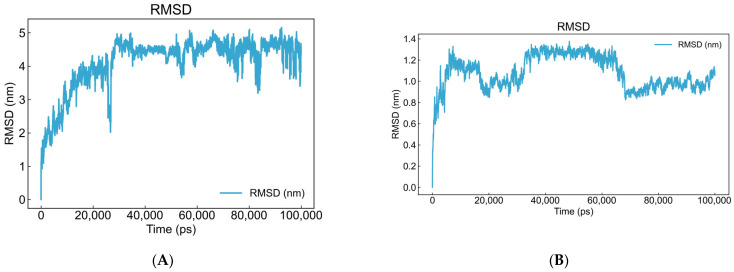
The variation in RMSD values of the BA and aptamer–BA complex over time. (**A**) The positional fluctuations of BA relative to its initial binding site within the DNA aptamer binding pocket. (**B**) The positional fluctuations of the aptamer–BA complex conformation relative to its initial site.

Figure 8A illustrates the positional fluctuations of BA relative to its initial binding site within the DNA aptamer binding pocket. The figure reveals a distinct rise and fluctuation in RMSD values between 0 and 30 ns. This represents a normal phenomenon, reflecting an initial relaxation and adjustment process as the ligand enters the binding pocket. The ligand interacts with surrounding DNA bases, seeking the most stable, lowest-energy binding conformation. After approximately 40 ns, the RMSD curve enters a flat, stable plateau. From a molecular perspective, this indicates that the position of BA has been “locked,” with virtually no significant translational or rotational movement.

To investigate the temporal evolution of the aptamer–BA complex conformation in the simulated system and its deviation from the initial state, we calculated the RMSD values of the complex. The results are shown in Figure 8B: Within 0–5 ns, the RMSD rapidly increased, followed by sustained fluctuations between 5–35 ns, indicating that the aptamer DNA structure was gradually relaxing from its initial high-energy state to a more stable conformation in the solvated environment. After approximately 35 ns, the RMSD first stabilizes, suggesting BA has been recognized and captured by the aptamer. The aptamer then undergoes rapid conformational adjustments to form a “binding pocket” accommodating BA, enabling optimal spatial alignment between the two for efficient interaction force establishment. By 65 ns, the RMSD enters a fully stable phase, indicating the formation of a stable complex structure between the aptamer and BA. The overall increasing trend in RMSD confirms that BA binding indeed induces significant conformational changes in the aptamer. The rapid stabilization of the system after approximately 35 ns further validates that the binding process occurs swiftly and efficiently reaches a binding equilibrium.

##### Analysis of Distance Changes Among Aptamer Bases G18, A20, G36, and G37

The RMSD analysis above indicates that BA binding induces significant structural changes in the aptamer binding site. To further investigate the specific pattern of this change, we monitored the dynamic evolution of distances between key base pairs (G18-A20, G18-G36, G18-G37, A20-G36, A20-G37, and G36-G37) over time.

**Figure 9:**
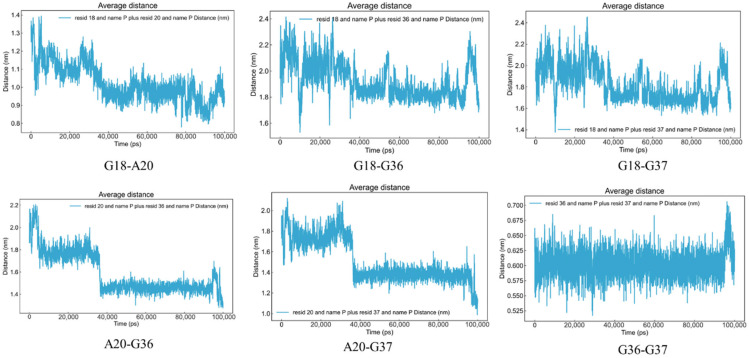
The variation in the distance between bases over time.

Figure 9 shows the MD simulation results, from which it can be seen that BA binding induces significant conformational rearrangement in the aptamer binding pocket, exhibiting a typical “induced fit” effect. During this process, key bases (G18, A20, G36, G37) approach each other to form a more compact and stable binding pocket, thereby better accommodating the ligand molecule.

G18, G36, and G37 were originally spatially distant from each other. The BA binding significantly narrowed the distance between them, causing the binding pocket to contract as a whole. This serves as direct evidence for the “induced fit” mechanism. Although the regions containing G36 and G37 exhibited some conformational fluctuations during binding, suggesting structural flexibility in this area, these fluctuations consistently followed the overall contraction trend, further confirming the systematic conformational adjustment of the binding pocket. The proximity of G18 to G36 and G37, along with changes in the distances between other bases, collectively reveal a “contraction-stabilization” binding mechanism involving the coordinated participation of multiple bases. Notably, the distance change curves between A20 and G36, as well as between A20 and G37, exhibit high consistency. Both curves show a marked decrease within the first 35 ns before stabilizing, a time point consistent with the equilibrium phase reached by the system in the aforementioned RMSD analysis. This result further supports the “induced fit” mechanism: BA binding triggers conformational adjustments in the aptamer, facilitating effective interactions between A20 and BA. This, in turn, drives the closure of the binding pocket, tightly encapsulating the BA molecule like a ‘lid’ or “clamp.” This conformational change represents a critical step in the binding process. In contrast, the distance between G18 and A20, as well as between G36 and G37, remained relatively stable throughout the simulation. This stability likely stems from their close spatial proximity and relatively rigid structural arrangement.

In summary, the dynamic changes in key base spacing clearly reveal the molecular recognition mechanism of BA aptamers: rather than passively accepting ligands, aptamers actively contract their binding pockets through coordinated conformational movements of key bases, optimizing their spatial structure to achieve efficient pairing with BA molecules. This ultimately forms a high-affinity, highly selective binding interface. These findings provide a theoretical basis at the atomic level for the exceptional binding properties of aptamers.

##### Analysis of the Number of Hydrogen Bonds Formed Between the Aptamer Bases G18, A20, G36, and G37 and BA

**Figure 10:**
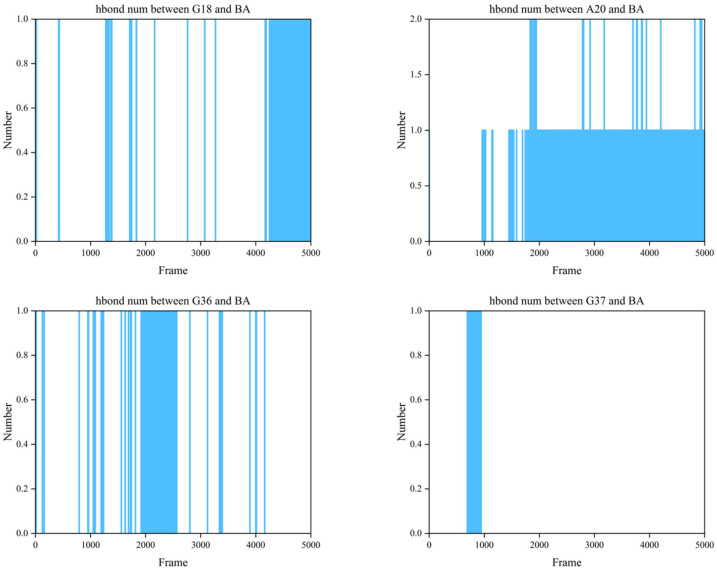
The number of hydrogen bonds formed by bases G18, A20, G36, and G37 with BA.

To investigate the binding mechanism between BA and the aptamer at the atomic level, this study analyzed the hydrogen bond formation between BA and four key bases (G18, A20, G36, G37) within the binding pocket during a 100 ns molecular dynamics simulation. The results are shown in Figure 10, with each frame representing 0.02 ns.

Figure 10 of the simulation results shows that different bases play completely different roles in hydrogen bond formation.

From the timing of hydrogen bond interactions between G18 and BA, it is primarily involved in the recognition and capture phase of the binding process. The hydrogen bond interaction between A20 and BA exhibits significant dynamic characteristics, continuously undergoing formation, breakage, and reformation throughout the simulation period. Notably, a stable hydrogen bond is observed at 40 ns, coinciding with the timing of changes in the base distances between A20-G36 and A20-G37. This indirectly demonstrates that the interaction between A20 and BA induces conformational changes in the aptamer. G36 exhibits distinct hydrogen bonding interactions with BA. Temporally, the formation of G36-BA hydrogen bonds precisely aligns with the aptamer’s conformational shift, particularly the bonds observed around 40 ns, which directly indicate G36’s involvement in BA recognition, capture, and subsequent aptamer conformational changes. G37 formed a hydrogen bond with BA before 20 ns, which subsequently broke. This timing aligns with the RMSD change in the aptamer–BA complex at 20 ns, confirming G37’s involvement in BA recognition and capture. This indirectly suggests that G37, by forming a highly stable hydrogen bond, bridges the distance between BA and the aptamer, thereby promoting the formation of the “induced fit” effect.

In summary, hydrogen bond analysis reveals that within the BA–aptamer complex, G18, G36, and G37 primarily participate in recognizing and capturing BA. Subsequently, A20 binds to BA, driving the conformational change in the aptamer to form a complementary, high-affinity binding interface.

### 3.3. Aptamer Application Performance Evaluation

**Figure 11:**
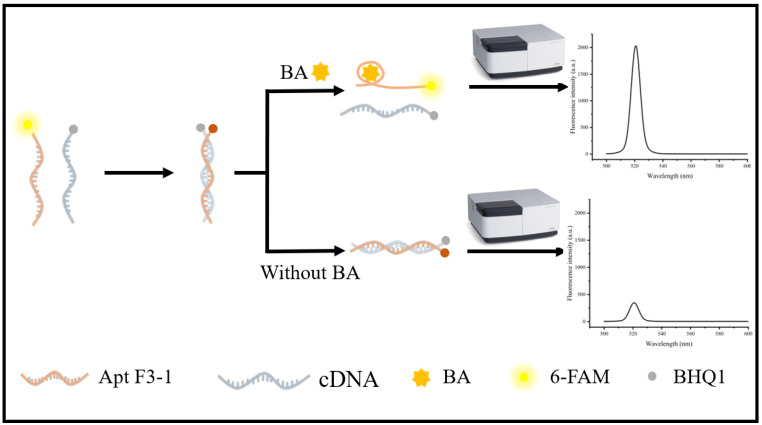
Schematic for detecting BA.

To validate the practical applicability of the high-affinity BA-specific aptamer F3-1, fluorescence-based assays were established for determination and specificity analysis. The specific detection principle is shown in Figure 11. The aptamer was labeled with 6-FAM at the 5′ end and its cDNA with BHQ1 at the 3′ end, constructing a 6-FAM–aptamer–BHQ1 duplex sensing system. In the absence of BA, the aptamer hybridizes with its complementary strand through Watson–Crick base pairing, bringing the fluorophore and quencher into close proximity and resulting in efficient fluorescence quenching. Upon addition of BA, the aptamer undergoes a conformational change to form a stable complex with BA, thus detaching from the complementary strand and restoring fluorescence. At low BA concentrations, the fluorescence signal increases steadily with increasing concentration due to gradual specific binding and progressive probe dissociation; at high concentrations, the signal tends to plateau, mainly ascribed to saturation of aptamer binding sites, maximum fluorescence recovery, as well as slight non-specific adsorption and self-absorption of the system, which jointly slow the response growth.

#### 3.3.1. Sensitivity and LOD

**Figure 12:**
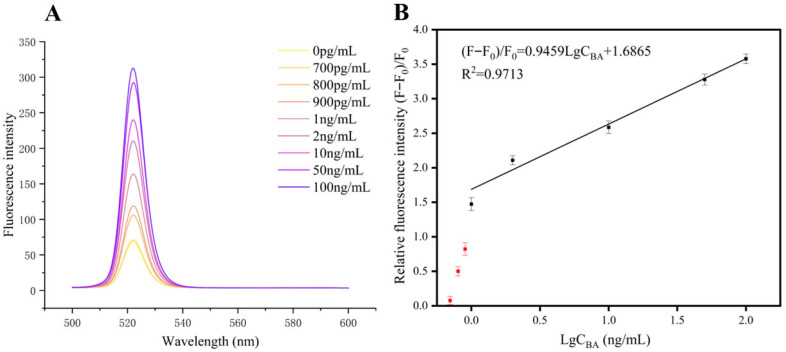
(**A**) Fluorescence intensity at 520 nm as a function of BA concentration (0–100 ng/mL). (**B**) Linear relationship between LgC_BA_ and (F − F_0_)/F_0_ in the range of 0.7–1 ng/mL.

As shown in Figure 12A, the fluorescence intensity was low without BA. Upon gradual BA addition, specific aptamer–BA binding caused a concentration-dependent increase in the sensor’s fluorescence signal, confirming the sensor’s capability for the quantitative detection of BA through fluorescence recovery. In the concentration range of 1–100 ng/mL, the relative fluorescence intensity (F − F_0_)/F_0_ exhibited a good linear relationship with the logarithm of BA concentration (Figure 12B).

#### 3.3.2. Specificity

**Figure 13:**
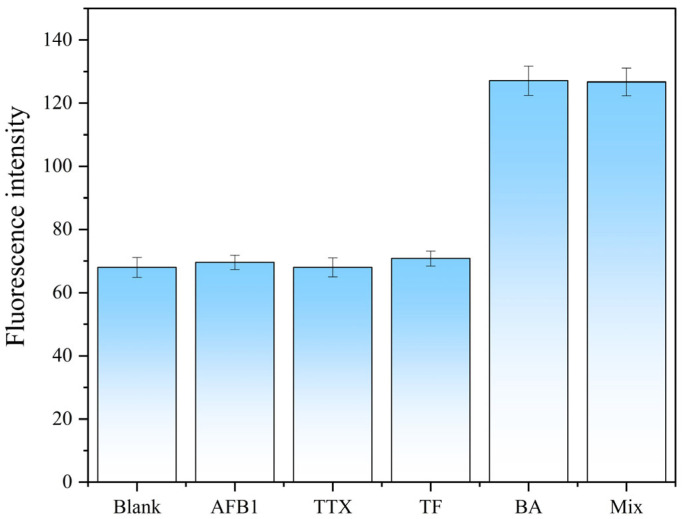
Specificity analysis of the F3-1 aptamer.

The specificity results are presented in Figure 13. The BA group and the mixed toxin group exhibited significantly higher fluorescence intensity compared to the blank group. In contrast, the interfering toxin groups (AFB1, TTX, TF) showed fluorescence intensities comparable to the blank group. Even at a concentration (10 ng/mL) that was 10-fold higher than the BA concentration (1 ng/mL), the interfering toxins did not induce significant fluorescence recovery. This indicates that the F3-1 aptamer specifically recognizes BA without cross-reactivity with the tested interfering toxins, confirming the excellent specificity of the aptamer and its potential for accurate BA detection in complex samples.

## 4. Conclusions

In this study, we successfully developed a high-performance DNA aptamer for BA and comprehensively elucidated its molecular recognition mechanism, with further validation of its recognition capability. Through SELEX screening, 12 BA-binding DNA aptamers were isolated, with F3 and F66 showing prominent binding activity (Kd 8.9 μM and 8.4 μM, respectively). Building on structural insights into aptamer–BA interactions, rational base substitution yielded the remodeled variant F3-1 with a Kd of 61.9 nM, which is 144-fold higher than that of the parental aptamer F3, marking the first reported DNA aptamer with nanomolar affinity for BA.

Beyond affinity improvement, our work provides a deep mechanistic understanding of the molecular recognition. MD simulations and hydrogen bond analysis demonstrated that G18, G36, and G37 function as core residues for initial BA recognition and capture, while A20 mediates subsequent conformational changes to stabilize the complex. This sequential binding process, coupled with the formation of specific hydrogen bonds and conformational rearrangement of the binding pocket, explains the high affinity and specificity of F3-1 for BA.

Although the selected aptamers exhibit high affinity and specificity toward BA, several limitations related to practical application should be noted. The binding performance of the DNA aptamer may be influenced by pH, ionic strength, and temperature, which affects its robustness under non-optimal conditions. Complex sample matrices may cause non-specific interactions and interfere with the specific binding of the aptamer. In addition, the aptamer may suffer from reduced activity during long-term storage or under harsh conditions without proper protection. Furthermore, as this study only focuses on aptamer development, no integrated, portable sensing device or simplified pretreatment strategy has been established, restricting direct field deployment. These issues are associated with subsequent sensor construction rather than the inherent properties of the aptamer and will be addressed in future application-oriented research.

Collectively, this work advances the field of BA-specific aptamer development and BA detection by identifying a panel of BA-binding DNA aptamers, engineering a high-affinity variant, deciphering the underlying induced-fit binding mechanism, and validating its recognition performance. These findings establish a robust foundation for the development of sensitive and specific BA detection platforms, with direct implications for safeguarding food safety and public health.

## Figures and Tables

**Table 1:** Asymmetric PCR reaction procedure and reaction system.

Reaction Procedure			Reaction System	
95 °C	5 min	/	2 × PCR Mix	25 μL
95 °C	30 s	30 cycles	10 μM Primer I	4 μL
56 °C	30 s		0.5 μM Primer II	4 μL
72 °C	30 s		ssDNA	2 μL
72 °C	5 min	/	ddH_2_O	5 μL

**Table 2:** DNA Sequences.

	Oligonucleotide Sequence (5′-3′)
F1	GACGATTCACTCCGCTAGCATTAATAAACGTGTTTCCCTT
F11	ACAACGTCGGTTCACACATATCCGGATATCTCGGAGAGCA
F2	GTTCACACGTCCCGTCTATCCGAATTTACAGACCTGAGAG
F22	GGCCAACCCTACCAGACTATGCCTTGTCGGAAACTGACCC
F21	CCACGAGACTCCGTTCGTATAGCAGTTCATTTGCGTAAAA
F3	GCCCGATGCGGGCAGGAGGCTGTATGGGCACCATTATTTG
F4	CGGAGTAGATGGTGGTACATCGGATCTATGCGTACCATGA
F5	TGTATCGCGGAGGGACTACGCAACACTAACGAGATATCAT
F55	GCCTGCATATGGAATTCGCCTTACCCCCGCCTATCGTTCG
F66	CCTGAATAAACTACCACTGACCGTCCCTTCCCTGAGTTAT
F1-1	AGGTAGACGCACATAGCACTCTGAAATAATATTCTGGTCG
F3-1′	CGTCGTTTTACAACGTCGTGACTGGGAAAACCCTGGCGTT
F3-1	GGCCGAAGCGGGCAGGAGGATGTGGGTCGACCAGAGGGGG
F3-2	GGCCGAAGCGGGCAGGAGGATGTGGGTCGACAAGAGGGGG
F3-3	GGCCGAAGCGGGCAGGAGGATGTGGGTCGACCAGAGGGAA
F3-4	GGCCGAAGCGGGCAGGAGGATGTGGGTCGACAAGAGGGAA
F3-5	GGCCGAAGCGGGCAGGAGGATGTGGGTCGACCAGAGGGGA
F3-6	GGCCGAAGCGGGCAGGAGGATGTGGGTCGACAAGAGGGGA
F3-7	GGCCGAAGCGGGCAGGAGGATGTGGGTCGACCAGAGGGAG
F3-8	GGCCGAAGCGGGCAGGAGGATGTGGGTCGACAAGAGGGAG
F66-1	CGTGAAAGAAGGACCAATGACCGGGCTCGCCCTGAGGGGG
F66-2	CGTGAAAGAAGGACCAATGACCGGGCTCGCCATGAGGGGG
F66-3	CGTGAAAGAAGGACCAATGACCGGGCTCGCCCTGAGGGAA
F66-4	CGTGAAAGAAGGACCAATGACCGGGCTCGCCATGAGGGAA
F66-5	CGTGAAAGAAGGACCAATGACCGGGCTCGCCCTGAGGGGA
F66-6	CGTGAAAGAAGGACCAATGACCGGGCTCGCCATGAGGGGA
F66-7	CGTGAAAGAAGGACCAATGACCGGGCTCGCCCTGAGGGAG
F66-8	CGTGAAAGAAGGACCAATGACCGGGCTCGCCATGAGGGAG
F22-1	GGCCAAAGCTGGCAGAATAAGCCGGGTCGGACAGAGGGGG
F22-2	GGCCAAAGCTGGCAGAATAAGCCGGGTCGGAAAGAGGGGG
F22-3	GGCCAAAGCTGGCAGAATAAGCCGGGTCGGACAGAGGGAA
F22-4	GGCCAAAGCTGGCAGAATAAGCCGGGTCGGAAAGAGGGAA
F22-5	GGCCAAAGCTGGCAGAATAAGCCGGGTCGGACAGAGGGGA
F22-6	GGCCAAAGCTGGCAGAATAAGCCGGGTCGGAAAGAGGGGA
F22-7	GGCCAAAGCTGGCAGAATAAGCCGGGTCGGACAGAGGGAG
F22-8	GGCCAAAGCTGGCAGAATAAGCCGGGTCGGAAAGAGGGAG
cDNA	CCCCCTCTGGTCGACCCACATCCTCCTGCCCGCTTCGGCC

**Table 3:** ITC tests thermodynamic parameters.

	Kd (μM)	*n*	ΔH (kJ/mol)	ΔS (J/mol·K)	ΔG(kJ/mol)
F1	92.71	0.748	−86.94	−214.4	−23.02
F2	75.51	0.705	−68.26	−150	−23.54
F22	45.86	0.915	−89.8	−218.1	−24.77
F3	8.927	0.422	−83.24	−182.5	−28.83
F55	28.81	0.63	−98.88	−244.7	−25.92
F66	8.406	0.779	−98.86	−234.4	−28.97

**Table 4:** Remoded aptamer ITC tests thermodynamic parameters.

	Kd (nM)	*n*	ΔH (kJ/mol)	ΔS (J/mol·K)	ΔG (kJ/mol)
F3-1	61.9	0.708	−93.18	−174.5	−41.18
F3-2	891.8	0.935	−95.77	−205.4	−34.56
F22-1	839	1.279	−94.89	−201.9	−34.72
F22-5	598.8	0.977	−81.58	−154.5	−35.54
F22-6	932.2	1.316	−40.15	−19.22	−34.42

## Data Availability

The original contributions presented in this study are included in the article/Supplementary Material. Further inquiries can be directed to the corresponding author.

## Supplementary Materials

The following supporting information can be downloaded at: https://www.mdpi.com/article/10.3390/foods15101663/s1, Figure S1: Secondary structures of the twelve aptamers.
