# Supplementary figures and images for: Study on the Modification and Minding Mechanism of Bongkrekic Acid Aptamers for Food Safety
Source: Foods. 2026 May 10;15(10):1663. doi: 10.3390/foods15101663 (PMC13205954; doi:10.3390/foods15101663)

Figure S1: Secondary structures of the twelve aptamers

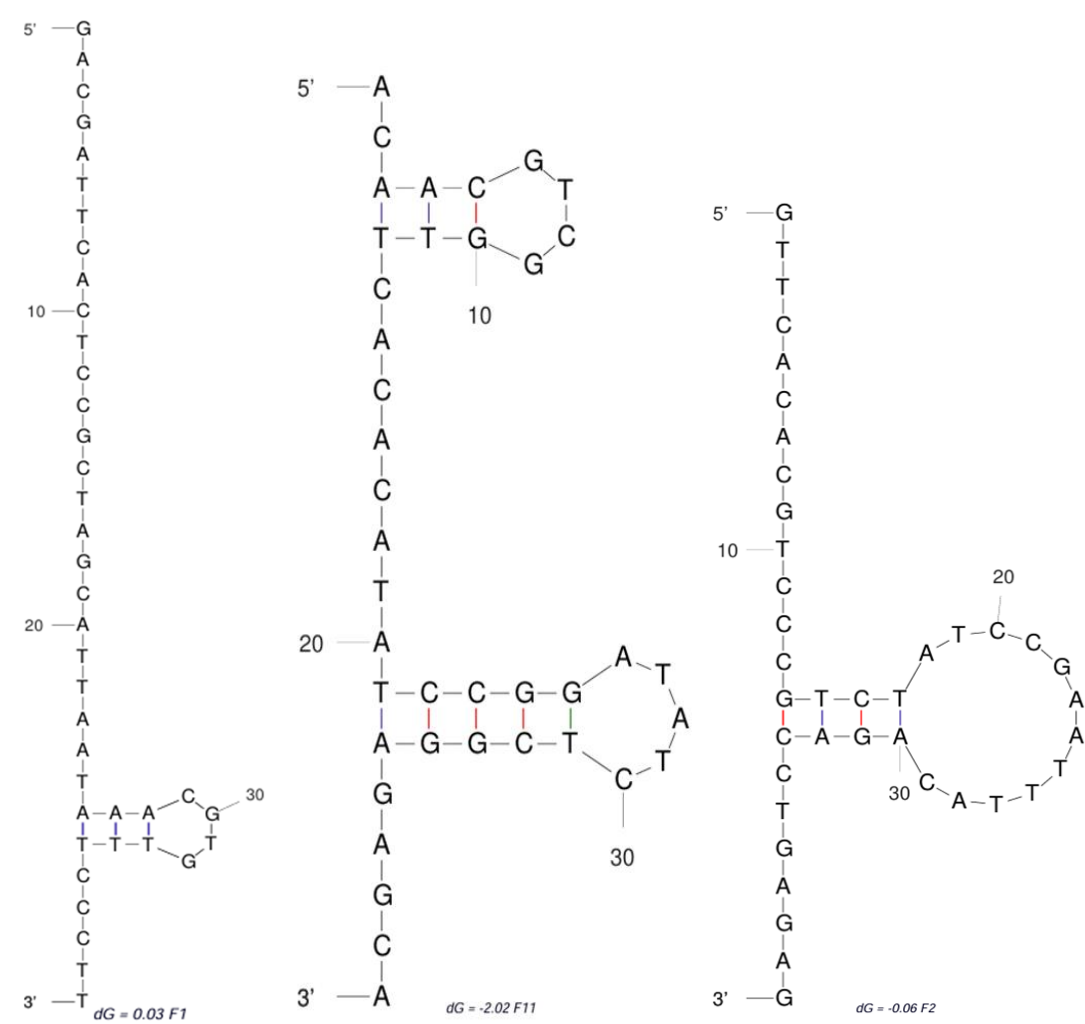

Supplement: Supplementary file 1 [file foods-15-01663-s001.zip › foods-4249902-supplementary.pdf]
